# Lifestyle diseases

**Published:** 2008-10

**479**

**Effects of advance drum drying processing of honey and garlic mix (*Madhu lasuna*)**

PK Perera^1^, A Bamunuarachchi^2^, KKDS Ranaweera^2^

**^1^University of Colombo; ^2^University of Sri Jayawardanapura.**

Mix of bee honey and garlic (in ayurvedic term *Madhu Lasuna*) is an Ayurvedic remedy which has cardiovascular curing effects (lipid/ cholesterol, blood pressure and fibrinolytic activity); chemoprevention; antimicrobial, antifungal, antiprotozoal, antioxidant activities and immunologic activity; how ever its use as *Madhu Lasuna* has been restricted due to difficulties in preparing it and less of keeping quality. Some individuals dislike the smell of garlic and are discouraged to consume garlic cloves kept in bee honey. The compounds of *Madhu Lasuna* mix, which also containing constituent of bee honey and garlic have been found by the chemical analysis carried out. Most of the active ingredients found have been clinically tested by early works. Therefore it is of important to develop a ready to use product which can be easily commercialized. A study was conduct to prepare dehydrated bee honey and garlic mix powder using an advanced drum drying technology and the formulation of novel dosage form (capsules) using the powder. From the medical point of view in dehydrated powder, major active substances from garlic (*Allium sativum*) play an important role in medicinal aspect. Chemical analysis of the *Madhu Lasuna* powder showed that concentrations of sulfur compounds vary enormously. The compounds were found in bee honey in the present study also plays an important major role giving health benefits to consumer. This powder was analyzed for its moisture, protein, fat fiber and ash content. The formulation was found to contain 3.87% moisture, 0.2% protein, 0.23% fat, 0.5213% of fiber, and 0.713% ash. Garlic in bee honey dehydrated powder was capsulated in hard gelatinized capsules. The capsules sealed in air tight in cellophane pouches were not deteriorated and the quality was kept high even after one year. According to the dose Bee honey and Garlic Mix capsules should be taken three a day as a prophylactic or two capsules three times a day when disease occurs.

**480**

**Lifestyle drugs in india: are we ready for them?**

Rahman S Z, Khan R A, Gupta V

**Jawaharlal Nehru Medical College, Aligarh Muslim University, Aligarh, India.**

**Introduction:**

‘Lifestyle drug’. is the drug that can modify or change a non-medical or non-health-related goal or a condition which is at the margin of health and well-being. It can be used to alter not only the appearance but also the physical and mental capabilities of the individual.

**Methods:**

The term ‘Lifestyle Drug’ is very difficult to define absolutely. Over the last few decades, “lifestyle drugs’ and ‘lifestyle medicines’ have been used with increasing frequency, but, no clear-cut definition and demarcation is ever suggested.

**Observation:**

The healthcare scenario in India has to face many challenges as we are lacking in a system like National Health Scheme (NHS) of UK or an equivalent. While 14% and 4% of health care payments in India are borne by government and insurance sectors respectively, 80% of the Indian population is spending money out-of-pocket on health sector. This could increase poverty by 2%. With Indian economy maturing benevolently at the rate of 8-9%, we have witnessed an altogether afresh India with a stupendous increase in the market of drug discovery and development. This has certainly made us vulnerable to issues related to ‘lifestyle drugs’

**Conclusion:**

Conclusion: There is a need to study the concept and impact of these drugs on society, particularly in India. India should focus more on ‘life saving’ and ‘essential medicines’, rather than ‘lifestyle drugs’. In a free market system, profits may be an indicator of what we want as individuals, but they may not be the best indication of what drugs we need as a society.

**481**

**Identification and characterization of potent PTP 1B inhibitor RBx BE8C2D81-001-001 from herbal extracts**

Rayasam Geetha Vani, Tulasi Vamshi Krishna, Sharma Sameer, Singh Yogendra, Shah Vanya, Davis Joseph Alex, Das Biswajit, Srinivas Kona Subrahmanya, Katiyar CK, Cliffe Ian, Ray Abhijit, Bhatnagar Pradip Kumar

**Department of Pharmacology, NDDR, RandD III, RANBAXY, Gurgaon. India.**

**Introduction:**

PTP 1B is a tyrosine phosphatase and functions as a negative regulator of insulin signaling by dephosphorylation of Insulin Receptor. Inhibitors of PTP 1B have been proposed to function as insulin sensitizers and have anti-diabetic function. Many herbal plants have been reported to have anti diabetic therapeutic properties.

**Materials and Methods:**

Purified PTP1B (Biomol) was used with either pNPP (Sigma) or DiFUMP as substrate for the in vitro enzyme assay. For enzyme kinetics the assays were performed using several substrate concentrations and inhibitor concentration. IR phopshorylation was monitored using western blotting with specific IR phosphorylated antibodies.

**Results:**

We have identified small molecule inhibitors of PTP 1B from Herbal extract. The inhibitor RBx BE8C2D81-001-001 inhibits PTP 1B with an IC50 of 252 nM in vitro. We demonstrate that RBx BE8C2D81- 001-001 exhibits a competitive and reversible mode of inhibition. In a cell based assay RBx BE8C2D81-001-001 also increases IR phosphorylation.

**Conclusion:**

These results demonstrate that RBx BE8C2D81-001-001 is a ‘bonafide’ PTP 1B inhibitor and functions in insulin signal transduction pathway.

**482**

**Identification of novel orally active dipeptidyl peptidase IV inhibitor 7E8E80B, equipotent and equi-efficaceous to Januvia^TM^**

Davis JA, Singh S, Roy S, Sundaram S, Sethi S, Benjamin B, Surender A, Khanna V, Mittra S, Pal C^1^, Mahajan D, Ahmed S, Sharma L, Rajivkant, Bansal VS, Saini KS, Sattigeri J, Palliwal J, Ray A, Bhatnagar PK

**New Drug Discovery Research, Ranbaxy Research Laboratories, Gurgaon, Haryana, India.**

**Introduction:**

The inhibition of DPPIV to increase the GLP-1 and insulin levels is a proven strategy to treat Type 2 diabetes mellitus. The present study describes the biological profile of a novel DPP-IV inhibitor, 7E8E80B.

**Methods:**

DPP-IV and other selectivity assays were performed using fluorogenic substrates with human recombinant or plasma enzyme. Ob/Ob mice (M/F), aging 4-6 weeks were fasted for 16 h followed by different doses of 7E8E80B or vehicle; 30 min. prior to oral glucose load (2 g/ kg). Blood samples were collected from tail vein at 0, 15, 30, 60 and 120 minutes. Plasma glucose, insulin and GLP-1 levels were measured using commercial kits. Male wistar rats (150-200 g) were used for the pharmacokinetic studies.

**Results:**

7E8E80B potently inhibited human plasma and recombinant DPP-IV enzyme (IC_50s_ 17 and 46 nM respectively). 7E8E80B is a competitive, reversible and slow-binding inhibitor of DPP-IV. It is selective over DPP8 (980 fold), DPP9 (2110 fold) and other DASH and non-DASH members. 7E8E80B (10 mg/kg) significantly reduced blood glucose AUC_(0-120min)_ in an OGTT comparable to Januvia^TM^ (19.4 vs. 19.2 %) and showed elevation of insulin (>2 fold) and GLP-1(4 fold) levels in ob/ob mice. It also inhibited DPP-IV activity > 40 % after 4 h with favourable PK profile: % *F* 55; C_max_ 790 ng/ml; t_1/2_ 1.6 h; CL_plasma_ 39.3 ml/min/ kg; V_ss_ 3.24 L/kg on i.v. administration. A good correlation between PK-PD profile was observed.

**Conclusion:**

7E8E80B is a potent, orally active inhibitor of human and rodent DPPIV.

**483**

**A random survey of use of herbal medicines as self treatment for diabetes mellitus and their potential for interactions with allopathic anti-diabetics**

Kaur N, Rai J

**Govt Medical College, Amritsar, Punjab, India.**

**Introduction:**

Diabetes mellitus is a chronic disorder and its incidence is on rise. The Indian culture is rich in herbal medicines and that is also true for diabetes mellitus. Most of the people take herbal medicines with prescribed antidiabetic drugs concurrently without informing their physicians and at times, undesirable interactions occur which often go undiagnosed.

**Objective:**

The aim of present study is to prevent interactions between herbal and allopathic medicines.

**Methods:**

A questionnaire based survey was conducted on the diabetic patients visiting Medicine OPD, Guru Nanak Dev Hospital, Amritsar.

**Results:**

Total 120 diabetic patients surveyed, 30% were taking herbal medicines as self-treatment. *Momordica charantia* (Bitter gourd, karela) was used by 27.6%, *Trigonella foenum-graecum* (fenugreek, methi) 33.2%, *Eugenia jambolana* (jamun) 11.1% and Ayurvedic drugs 27.6%. 50% of diabetic patients taking unofficial preparations (Desi dvai) were unable to tell about the contents. 41.6% patients admitted that they will continue self treatment with prescribed drugs and 66.7% patients admitted of not informing physicians about self-treatment. 43% patients opined herbal medicines as safe and 33.3% as easily available.

**Conclusion:**

In literature, cases of hypoglycaemia due to concurrent use of *Momardica charantia* and chlorpropamide have been reported. Death due to hypoglycaemia has also been reported in diabetic patient on antidiabetic drugs with concurrent use of herbal drugs for psoriasis. Thus before prescribing antidiabetic drugs physicians should advise their patients to avoid concurrent use of herbal medicines to avoid undesirable interactions and ensure safety of prescribed treatment.

**484**

**Lithium chloride protects vascular dysfunction in high fat fed STZ diabetic rats**

Thakkar JH, Kanzariya NR, Patel Rameshvar K, Patel NJ

**S.K.Patel College of Pharmaceutical Education and Research, Ganpat University, Gujarat, India.**

Untreated type-II diabetes may leads to various vascular complications. We aimed to determine whether lithium chloride treatment impaired the vascular risk development in diabetic rats or not. Diabetes was produced by high fat diet and low dose of streptozotocin. After 2 week of high fat fed all the male S.D. rats were injected with STZ (35 mg/kg. i.p.). After six week of STZ administration diabetic rats were sacrificed under mild anesthesia and thoracic aorta was isolated. Contractile response of H_2_0_2_ (10^-6^ M to 10^-3^M) in control and diabetic rat thoracic aorta was measured on multi channel data acquisition system Poworlab/SSP (AD Instruments. Australia). Lithium chloride treatment was given *in-vitro* in concentration of 0.6, 5 and 20 mM. In *in-vivo* study, lithium chloride treatment was given in various dosages like 5, 25 and 50 mg/kg for four weeks. Various biochemical parameters like blood glucose, insulin, triglyceride, Cholesterol and antioxidant parameters were measured. Four week lithium chloride treatment showed significant increase in SOD, Catalase and decrease in MDA levels, indicate anti-oxidant activity. Lithium chloride treatment did not normalize the blood glucose, insulin, triglyceride and cholesterol levels. Contractile responses of H_2_O_2_ were increased in diabetic animals compared to normal control animals. While treatment with lithium chloride showed the decrease in H_2_O_2_ contractility in rat thoracic aorta in concentration dependent manner in *in-vitro* and *in-vivo* also. From this results it is concluded that lithium chloride treatment inhibit the diabetes induced vascular dysfunction.

**485**

**Survey of awareness of diet, drugs and complications in patients of diabetes mellitus in tertiary care hospital**

Kumar H, Goel A, Rai J, Bedi S

**Government Medical College, Amritsar, India.**

**Introduction:**

Diabetes mellitus is a multisystem disorder with an increasing incidence in developing countries like India. Awareness among people about diabetes is essential to ensure success and safety of drug treatment.

**Objective:**

The aim of the present study is to know the awareness level and make people aware about the diabetes.

**Methods:**

A questionnaire based survey has been conducted on 100 diabetic patients visiting Diabetic Clinic and admitted at Guru Nanak Dev Hospital, Amritsar. The questions asked inciude duration of diabetes, frequency of blood sugar estimation, dietary and exercise habits, medication used, missing doses of medicines and the organs affected by diabetes. 60 patients were females and 40 males belonging to age ranging from 40 to 75 years. 60% patients knew about the disease and duration was more than four years. Fasting blood sugar was being tested at weekly to two monthly intervals. 40% were aware about disease and duration of two weeks to two years and getting FBS tested occasionally. 66% were using sweets and 56% were taking all fruits. Exercise was done by 24% patients. 79% patients were taking oral antidiabetic drugs and 21% patients were on insulin. 54% were not taking drugs regularly and were not aware of its complications. 60% did not know about organs affected by diabetes.

**Conclusion:**

Present data suggests that while prescribing antidiabetics, patients should also be advised about life style modifications and regular use of medicines.

**486**

**Effect of *Trigonella foenum* (methi) on fasting blood glucose levels in alloxan induced diabetes in rabbits**

Subhani G, Shantamma

**Kamineni Instiute of Medical Sciences, Narketpally, India.**

**Introduction:**

Antidiabetic plant medicines might provide an important source of new oral hypoglycemic compounds for development as pharmaceutical entities, or as simple dietary adjuncts to existing therapies. Powdered fenugreek seeds when added to an oral glucose tolerance test solution significantly reduced postprandial glucose levels in experimentally induced diabetic rabbits.

**Aims and Objectives:**

To study the effect of the alcohol extract of seeds of *Trigonella foenum* (methi)on fasting blood sugar in alloxan-induced diabetes in rabbits.

**Materials and Methods:**

Alcoholic extract of fenugreek seeds was prepared by cold percolation method by using a soxhlet apparatus. 30 rabbits were induced with diabetes with alloxan.Fasting blood glucose levels were measured. They were divided into 5 groups: group1- control (gum acasia 2%) group 2 - metformin (62.5mg /kg) group 3 - fenugreek extract in graded doses(per kg bodyweight) 0.5gm, 1gm and 1.5gm. Rabbits with blood sugar 220-500mg/dl were considered diabetic. Follow-up study was done for 35 days and fasting blood sugar levels were recorded.

**Results:**

The study showed hypoglycemic effect of the extract in the oral dose range of 0.5 to 1.5 gm / kg body weight of rabbits.The hypoglycemic effect was comparable to that of established antidiabetic drug Metformin in the dose of 62.5 mg / Kg.

**Conclusion:**

fenugreek extract has shown significant reduction in fasting blood glucose levels in diabetic rabbits. They may help to reduce the dosage of conventional antidiabetic agents and may potentate their effect when given as a dietary adjunct.The broad dose range of the extract producing hypoglycemic effect in diabetic rabbit was an interesting observation, which requires further study.

